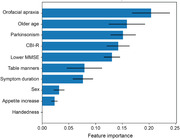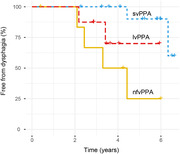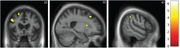# Dysphagia in primary progressive aphasia: clinical predictors and neuroanatomical basis

**DOI:** 10.1002/alz.089042

**Published:** 2025-01-09

**Authors:** Salvatore Mazzeo, Eoin Mulroy, Jessica Jiang, Michael Lassi, Jeremy Johnson, Chris JD Hardy, Jonathan D. Rohrer, Jason D Warren, Anna Volkmer

**Affiliations:** ^1^ Dementia Research Centre, UCL Queen Square Institute of Neurology, University College London, London United Kingdom; ^2^ Vita‐Salute San Raffele University, Milan Italy; ^3^ Department of Neuroscience, Psychology, Drug Research and Child Health, University of Florence, Florence Italy; ^4^ Dementia Research Centre, Department of Neurodegenerative Disease, UCL Queen Square Institute of Neurology, University College London, London United Kingdom; ^5^ The BioRobotics Institute and Department of Excellence in Robotics and AI, Scuola Superiore Sant’Anna, Pisa, Pisa Italy; ^6^ Department of Psychology & Language Sciences, University College London, London United Kingdom

## Abstract

**Background:**

Dysphagia is an important feature of neurodegenerative diseases and potentially life‐threatening in primary progressive aphasia (PPA), but remains poorly characterised in these syndromes. We hypothesised that dysphagia would be more prevalent in nonfluent/agrammatic variant (nfv)PPA than other PPA syndromes, predicted by accompanying motor features and associated with atrophy affecting regions implicated in swallowing control.

**Methods:**

In a retrospective case‐control study at our tertiary referral centre, we recruited 56 patients with PPA (21 nfvPPA, 22 semantic variant (sv)PPA, 13 logopenic variant (lv)PPA). Using a proforma based on caregiver surveys and clinical records we documented dysphagia (present/absent) and associated, potentially predictive clinical, cognitive and behavioural features. These were used to train a machine learning model. Patients’ brain MRI scans were assessed using voxel‐based morphometry and region‐of‐interest analyses comparing differential atrophy profiles associated with dysphagia presence/absence.

**Results:**

Dysphagia was significantly more prevalent in nfvPPA (43% vs 5% svPPA and no lvPPA). The machine learning model revealed a hierarchy of features predicting dysphagia in the nfvPPA group, with excellent classification accuracy (90.5% [95% confidence interval 77.9:100]): the strongest predictor was orofacial apraxia, followed by older age, parkinsonism, more severe behavioural disturbance and more severe cognitive impairment. Significant grey matter atrophy correlates of dysphagia in nfvPPA were identified in left middle frontal, right superior frontal and right supramarginal gyri, and right caudate.

**Conclusion:**

Dysphagia is a common feature of nfvPPA, linked to underlying cortico‐subcortical network dysfunction. Clinicians should anticipate this symptom particularly in the context of other motor features and more severe disease.